# *In Vivo* Diagnostic Imaging Using Micro-CT: Sequential and Comparative Evaluation of Rodent Models for Hepatic/Brain Ischemia and Stroke

**DOI:** 10.1371/journal.pone.0032342

**Published:** 2012-02-23

**Authors:** Naoto Hayasaka, Nobuo Nagai, Naoyuki Kawao, Atsuko Niwa, Yoshichika Yoshioka, Yuki Mori, Hiroshi Shigeta, Nobuo Kashiwagi, Masaaki Miyazawa, Takao Satou, Hideaki Higashino, Osamu Matsuo, Takamichi Murakami

**Affiliations:** 1 Department of Anatomy and Neurobiology, Kinki University School of Medicine, Osaka-Sayama, Osaka, Japan; 2 Department of Physiology, Kinki University School of Medicine, Osaka-Sayama, Osaka, Japan; 3 Department of Pharmacology, Kinki University School of Medicine, Osaka-Sayama, Osaka, Japan; 4 Biofunctional Imaging Laboratory, Immunology Frontier Research Center, Osaka University, Suita, Osaka, Japan; 5 Hitachi Aloka Medical Co., Ltd., Suita, Osaka, Japan; 6 Department of Radiology, Kinki University School of Medicine, Osaka-Sayama, Osaka, Japan; 7 Department of Immunology, Kinki University School of Medicine, Osaka-Sayama, Osaka, Japan; 8 Department of Pathology, Kinki University School of Medicine, Osaka-Sayama, Osaka, Japan; 9 PRESTO, Japan Science and Technology Agency (JST), Kawaguchi, Saitama, Japan; University of South Florida, United States of America

## Abstract

**Background:**

There is an increasing need for animal disease models for pathophysiological research and efficient drug screening. However, one of the technical barriers to the effective use of the models is the difficulty of non-invasive and sequential monitoring of the same animals. Micro-CT is a powerful tool for serial diagnostic imaging of animal models. However, soft tissue contrast resolution, particularly in the brain, is insufficient for detailed analysis, unlike the current applications of CT in the clinical arena. We address the soft tissue contrast resolution issue in this report.

**Methodology:**

We performed contrast-enhanced CT (CECT) on mouse models of experimental cerebral infarction and hepatic ischemia. Pathological changes in each lesion were quantified for two weeks by measuring the lesion volume or the ratio of high attenuation area (%HAA), indicative of increased vascular permeability. We also compared brain images of stroke rats and ischemic mice acquired with micro-CT to those acquired with 11.7-T micro-MRI. Histopathological analysis was performed to confirm the diagnosis by CECT.

**Principal Findings:**

In the models of cerebral infarction, vascular permeability was increased from three days through one week after surgical initiation, which was also confirmed by Evans blue dye leakage. Measurement of volume and %HAA of the liver lesions demonstrated differences in the recovery process between mice with distinct genetic backgrounds. Comparison of CT and MR images acquired from the same stroke rats or ischemic mice indicated that accuracy of volumetric measurement, as well as spatial and contrast resolutions of CT images, was comparable to that obtained with MRI. The imaging results were also consistent with the histological data.

**Conclusions:**

This study demonstrates that the CECT scanning method is useful in rodents for both quantitative and qualitative evaluations of pathologic lesions in tissues/organs including the brain, and is also suitable for longitudinal observation of the same animals.

## Introduction

Animal models of pathological states, including genetically engineered animals such as transgenic and knockout mice, as well as induced and spontaneous disease models, are valuable for mechanistic studies of similar disorders in humans. These animal models are also useful for the rapid screening of pharmaceuticals for action on a target specific to the disease being studied [1]. Until recently, such studies have been labor-intensive because it is often necessary to sacrifice significant numbers of animals under different experimental conditions to fully diagnose their pathology, severity, and rate of progression or remission. Therefore, there has been a growing need to develop new non-destructive technologies for more efficient studies with the animal models.

We have previously studied several pathological rodent models and reported their novel characteristics relevant to the human diseases they model. These include stroke-prone spontaneously hypertensive rats (SHRSP) [2], PIT-BD (photochemically induced thrombotic brain damage) mice [3], MCAO (middle cerebral artery occlusion) mice [4], and PIT-LD (photochemically induced thrombotic liver damage) mice [5]. These animal models have been useful respectively for studying human spontaneous stroke (cerebral infarction and/or hemorrhage), ischemic brain damage, cerebral infarction, and ischemic liver injury. The main problems with previous studies, including ours, however, were the labor-intensiveness of the experimental procedures and the difficulty in precise volumetric measurements. In our previous studies, for instance, surgical manipulation to create the model was required on dozens of animals for each experiment, and the brains or livers of several treated animals and matched controls were sampled at defined time-points after surgical induction of the disease (e.g., on days 1, 4, 7 and 14). The organs from each animal were subsequently fixed, sectioned, stained, and examined histologically to measure the injured area of each organ from its serial sections [3], [6]. The lesion volumes were roughly estimated from the lesion area and thickness of each section. These widely used protocols for histopathological studies have advantages for detailed analysis of the animal models through further application of histochemistry and immunohistochemical staining. However, for studies in which a specific treatment/surgery is needed for each animal and/or individual variation has to be accounted for, this approach could be disadvantageous because of its inefficiency, difficulty in collecting sufficient numbers of samples, and inaccuracy.

Recent advances in imaging methods in clinical settings, such as computed tomography (CT), magnetic resonance imaging (MRI), and positron emission tomography (PET), have triggered the extension of these technologies to studies of experimental animals [1]. Nonetheless, these methods are not yet been commonly used with experimental animals because of their significant costs and poorer performance in comparison with corresponding clinical equipment. However, the recently developed micro-CT scan can be a cost-effective technique that provides high-resolution images and rapid data acquisition from experimental animals, although its use is limited by its relatively poor soft tissue contrast as compared to human CT or other imaging techniques [1], [7], [8]. Therefore, until recently, application of micro-CT imaging to small experimental animals has been primarily restricted to skeletal structures and adipose tissues. Further, while contrast-enhanced CT imaging of organs such as liver has improved with the development of the micro-CT scanner, specific contrast agents and scan protocols [9], [10], *in vivo* micro-CT imaging of the brain remains a challenge because contrast resolution of the brain is much lower than that of other organs [11]. Recently, several *ex vivo* studies on cerebral vasculature and anatomy of mice and rats, including one using isolated MCAO rat brain [12], have demonstrated high spatial resolution and signal-to-noise ratio [13], [14], [15]. Although a few *in vivo* trials of micro-CT imaging in brain have reported acquisition of contrast-enhanced images of injected blood or xenografted glioma [16], [17], high-quality imaging for advanced studies such as diagnostic imaging or sequential study of animal disease models has not yet been achieved.

The aims of this study were to examine whether i) brain CECT is useful as a non-invasive method with high resolution and accuracy for research on rodent disease models, ii) CECT for small animals is applicable for studies such as serial observation of the same animals and/or diagnosis of lesions in spontaneous disease models, and iii) CECT could potentially be an alternative to conventional histological approaches for diagnosis. To this end, we employed a new micro-CT instrument in combination with continuous administration of an iodine contrast agent, and optimized CECT protocols for image acquisition in four different disease models.

## Materials and Methods

### Animals

Male inbred BALB/c mice (Japan Crea) were purchased and used for the generation of the MCAO and PIT-BD models. The plasminogen knockout mice (*Plg* −/−) and their wild-type littermates (*Plg* +/+) with mixed backgrounds of C57BL/6J (75%) and 129/SvJ (25%) were used for generating the PIT-LD models [5], [6]. Male SHRSP/Kpo rats (Experimental Animal Facility at Kinki University School of Medicine) were used as a spontaneous stroke model. All animals were cared for in accordance with the Law (No. 105) and Notification (No. 6) of the Japanese government, and all experiments were conducted with the permissions of the Experimental Animal Welfare Committees of Kinki University (KAME019-039, KAME19-026, KAME-19-047 and KAME-19-050) and Osaka University (FBS 07-001).

### Animal disease models

The MCAO, PIT-BD and PIT-LD models were generated as previously described [3], [6]. Briefly, these mouse models were generated as follows.

#### The MCAO model

Under 2% isoflurane anesthesia, the skull was exposed and a small opening was made in the upper region of the zygomatic arch with a handheld drill. The middle cerebral artery was occluded by ligation with nylon thread (10-0; Johnson and Johnson).

#### The Photochemically Induced Thrombotic (PIT) models

The PIT models (PIT-BD and PIT-LD, as described below) are ischemic injury models induced by *in vivo* administration of a photo-reactive dye (Rose Bengal) followed by the activation of the dye in a selected area of the brain or liver by green light irradiation resulting in the generation of reactive oxygen species, which lead to thrombosis in the area adjacent to the light source [3], [6], [18], [19].

i) *The PIT-BD model:* Under isoflurane anesthesia, a cannula was inserted into the jugular vein. The skin on the top of the head was incised, and an optic fiber (1.0 mm in diameter) was placed on the skull. Immediately after the infusion of 20 mg/kg Rose Bengal (Wako Pure Chemicals) through the cannula, the brain area adjacent to the optic fiber was illuminated with green light (wavelength, 540 nm; illumination intensity, 4.7×10^−4^ W/cm^2^) for 10 min using a heat-absorbing light source (model L5178, Hamamatsu Photonics). After illumination, the incised skin was sutured under sterile conditions and the cannula was withdrawn.

ii) *The PIT-LD model*: The left lateral liver lobe was exposed after laparotomy under isoflurane anesthesia, and an optic fiber (3 mm in diameter) was allowed to gently touch the center of the lobe. The right jugular vein was cannulated with a polyethylene tube containing saline and connected to a microinjection pump. Rose Bengal (10 mg/kg) dissolved in saline was infused at a rate of 50 µl/min followed immediately by illumination with green light (wavelength, 540 nm; illumination intensity, 2.36×10^−2^ W/cm^2^) for 10 min by using the above light source (model L5178).

#### The human stroke or hypertensive encephalopathy model

The SHRSP/Kpo rat was used as a spontaneous model of human stroke or hypertensive encephalopathy [20]–[22]. Systolic blood pressure (SBP) and heart rate were measured by tail-cuff plethysmography (UR-5000; Elquest) once a week throughout the experiments. Typically, SBP was over 230 mm Hg in the SHRSP rats. Body weight was measured every day. Stroke incidence was estimated based on blood pressure and heart rate measurements, body weight, and neurological symptoms as described previously [23].

### Contrast enhanced micro-CT (CECT) imaging

*In vivo* CECT imaging was performed with an X-ray CT system (Latheta LCT-200, Hitachi Aloka Medical) newly developed for imaging small experimental animals. Each experiment included 3–5 animals (see Results). Mice were anesthetized with isoflurane, and Iohexol, an iodine contrast medium with a concentration of 300 mg iodine/ml (mg I/ml; Daiichi-Sankyou, Tokyo), was infused intravenously at a rate of 5 ml/hr for 5 min before CT scanning, followed by a continuous infusion rate of 1 ml/hr during the entire CT examination. For rats, the infusion rate was 30 ml/hr for 5 min before the CT scan, followed by a continuous infusion rate of 6 ml/hr during image acquisition. Parameters used for the CT scans were as follows: tube voltage: 50 kV; tube current: 0.5 mA; axial field of view (FOV): 60 mm for rats and 48 mm for mice, with an inplane spatial resolution of 48 µm×48 µm and slice thickness of 192 µm for the liver and 384 µm for the brain. Images were generated by integration of four and eight signal averages, respectively, for the liver and brain, and image acquisition for the liver was respiratory-gated. Total scan time was approximately 15 min for the liver and 20 min for the brain. Quantitative assessment of the lesion area was performed with LaTheta software (version 3.00). Three-dimensional CT pictures of the brain and liver were reconstructed with the VGStudio MAX2.0 software (Nihon Visual Science, Tokyo, Japan). To diagnose and analyze brain lesions of the MCAO, PIT-BD, and SHRSP models by CECT, high attenuation area (HAA) was set at CT values higher than 150, and low attenuation area (LAA) at CT values less than 80, based on the average CT values obtained from the acquired images. HAA at CT values of higher than 450, and LAA at those of less than 300 were used for measuring injury in PIT-LD mice.

### Micro-magnetic resonance imaging (micro-MRI)

An MRI apparatus for small animals at 11.7 T (Bruker, AVANCE 500WB) was used for the experiments. Rats (SHRSP, n = 5) or mice (MCAO, PIT-BD and PIT-LD, n = 4 each) were anesthetized with isoflurane and MRI was performed under the following conditions:

- For the rat study, T2-weighted fast spin echo (Rapid Acquisition with Relaxation Enhancement: RARE) sequence (TR/TE = 6000/45 ms; FOV = 25×25 mm; matrix size = 256×256; slice thickness = 0.5 mm; NS = 16; total scan time = 25 min approx.) and T1-weighted gradient echo (Fast Low Angle Shot: FLASH) sequence (TR/TE = 300/2.6 ms; FOV = 25×25 mm; matrix size = 256×256; slice thickness = 0.5 mm; NS = 8; total scan time = 6 min approx.) were used.
- For the mouse brain study, T2-weighted fast spin echo (RARE) sequence (TR/TE = 5000/35.4 ms; FOV = 25×25 mm; matrix size = 256×256; slice thickness = 0.5 mm; NS = 8; total scan time = 10 min approx.) was used.
- For the mouse liver study, T2-weighted fast spin echo (RARE) sequence (TR/TE = 5000/36 ms; FOV = 20×20 mm; matrix size = 256×256; slice thickness = 0.5 mm; NS = 8; total scan time = 10 min approx.) was used.

### Histology

Following the CT scan or MRI, mice and rats were anesthetized and perfused with 4% paraformaldehyde. The brain or liver was removed, embedded in paraffin, sectioned, and used for histological analyses. Sections were stained with hematoxylin and eosin (H&E) according to standard protocols. For the detection of ischemia, 2% 2,3,5-triphenyltetrazolium chloride (TTC) staining was performed on the brain sections (1-mm thickness).

### Assessment of vascular permeability

Blood-brain barrier permeability was measured using Evans blue dye leakage as described elsewhere [24]. Briefly, after CECT and MR scans were performed on the MCAO and PIT-BD mice (n = 5 each), 200 mg/kg body weight of a 4% solution of Evans blue in saline was injected intravenously. 90 min after injection, animals were sacrificed and were perfused with 20 ml of saline through the left heart. The brain was removed and sectioned in 2- (MCAO) or 3-mm (PIT-BD) segments. Then all slices were sectioned in the middle, and ipsilateral (including the ischemic area) and contralateral (control) segments were collected separately. Formamide (300 µl) was added in each tube containing the tissue sections and incubated at 55°C for 72 h to extract Evans blue dye. The absorbance at 620 nm of the supernatant was measured and the amount of Evans blue in each hemisphere was calculated from a standard curve. The difference between the ipsilateral and contralateral hemispheres was considered to represent the amount of extravasated Evans blue in the lesion.

### Statistics

**Figure 1:**
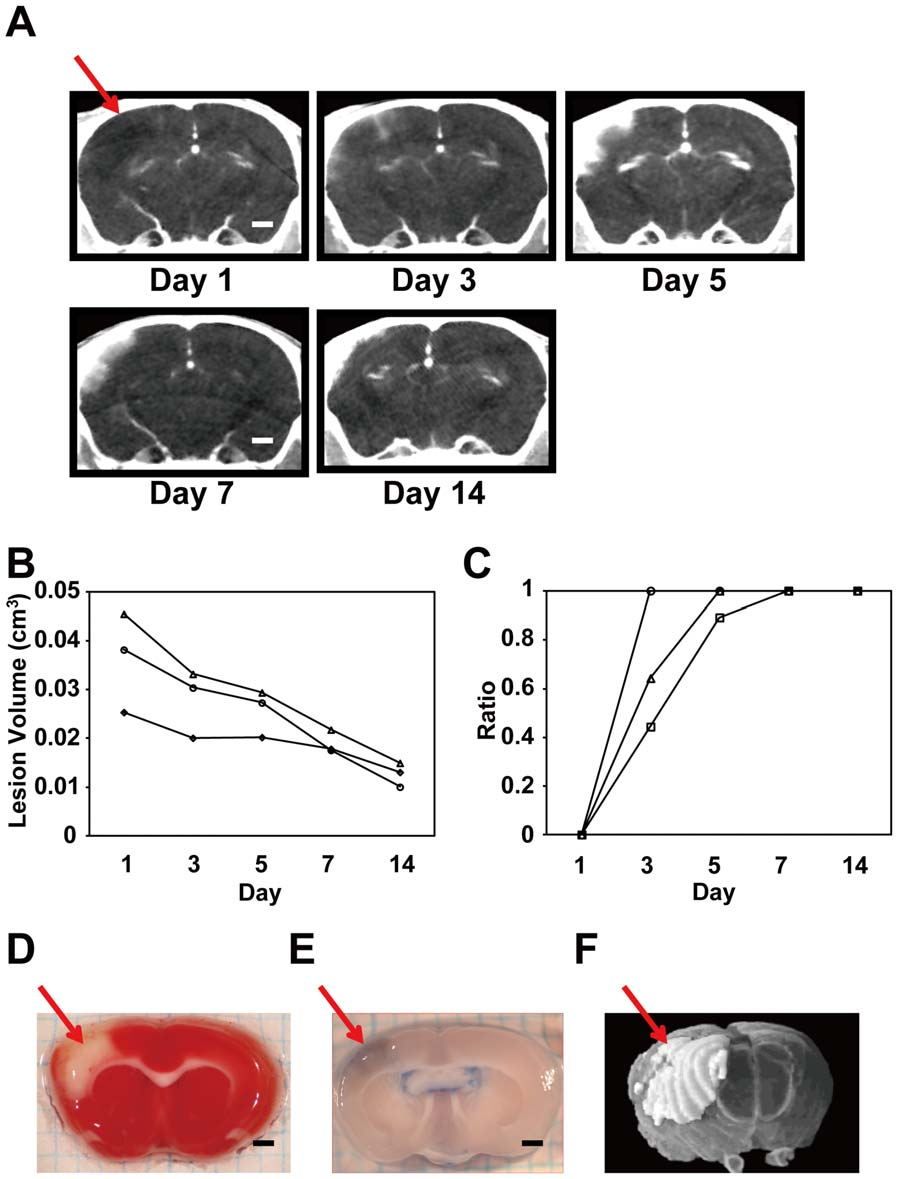
Temporal changes in cerebral lesions in the MCAO mouse infarction model. **A.** Representative serial CECT images acquired from the same MCAO mouse brain scanned over a two week period. The red arrow indicates the infarct region. **B.** Quantitative changes in the lesion area of the MCAO brain over time. Changes in lesion volumes of three MCAO mice through days 1 and 14 were plotted. **C.** Progressive changes in the ratio of the HAA (%HAA) in the same MCAO mice shown in B. **D.** A coronal brain slice of an MCAO mouse on day 7 stained with TTC. The ischemic lesion is indicated by the arrow. **E.** Increased vascular permeability in the lesion shown by Evans blue leakage. **F.** A three-dimensional (3-D) image of the infarct area in an MCAO brain (white area, arrow) reconstructed from sequential coronal CECT images. All scale bars indicate 1 mm (A, D, E).

**Figure 2 pone-0032342-g002:**
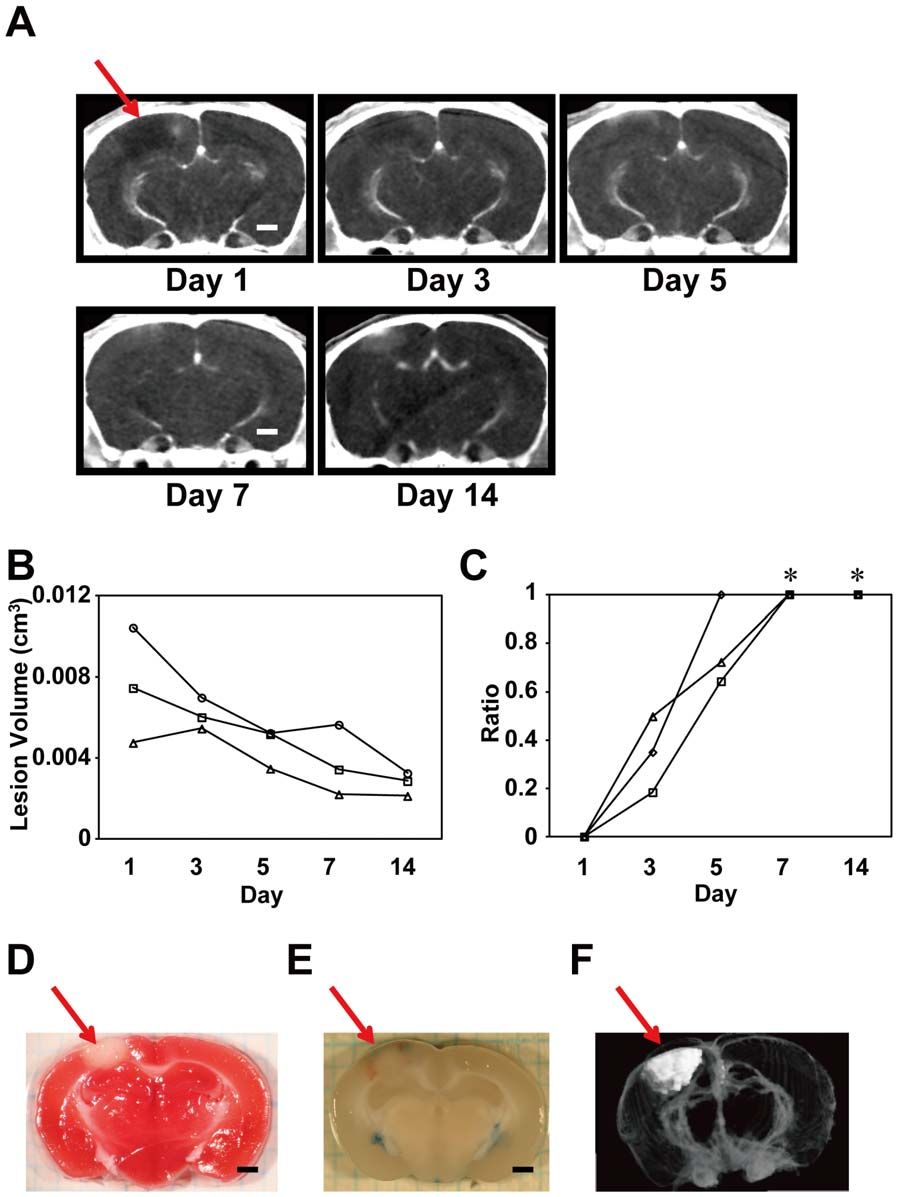
Temporal changes in the infarct area in the PIT-BD mouse model. **A.** Serial CECT images taken from a representative PIT-BD mouse brain. **B.** Time-dependent changes in %HAA and total volume of lesion. Changes in lesion volumes of three MCAO mice through days 1 and 14 are shown. **C.** Temporal changes in %HAA in the same PIT-BD mice shown in B. There were significant differences in %HAA between day 1 and days 7 and 14 (Kruskal-Wallis test with Dunn's post-hoc tests, *P<0.05). **D.** A TTC-stained coronal section of a PIT-BD mouse brain on day 7. The arrow indicates the ischemic lesion. **E.** Leakage of Evans blue indicating elevated vascular permeability in the infarct area. **F.** A 3-D image of the PIT-BD lesion (white area) reconstructed from sequential CECT images. Scale bars: 1 mm (A, D, E).

**Figure 3:**
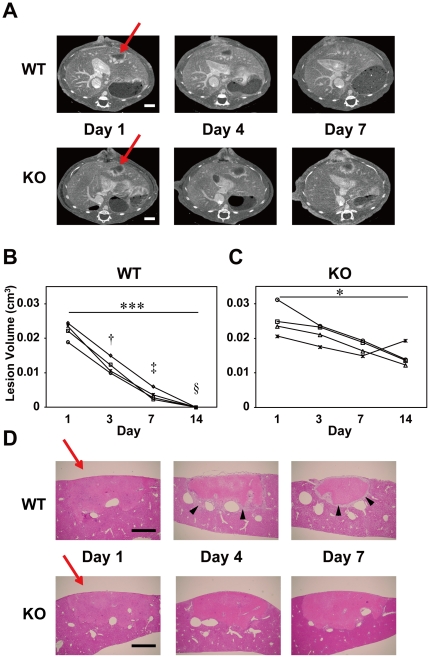
Differences in the temporal changes in liver lesions between the WT and plasminogen KO mice. **A.** CECT scans showing time-dependent recovery from PIT-LD liver injury in wild-type (WT), but not in the plasminogen-knockout (KO) mouse. The arrows indicate areas of photochemically induced injury. Scale bars indicate 2 mm. **B.** Quantitative evaluation of the recovery process from the liver damage in the WT mice. Changes in lesion volumes in WT PIT-BD mice from day 1 through day 14 are shown (n = 4). Kruskal-Wallis test with Dunn's post-hoc tests indicated a significant difference in the lesion volumes between days 1 and 14 (***, *p*<0.001). Two-way ANOVA with Bonferroni post-hoc tests revealed significant differences in the lesion volumes between WT and KO mice on days 4 (†, *P*<0.001), 7 (‡, *P*<0.0001) and 14 (§, *P*<0.0001). **C.** Changes in the lesion volume in livers of the plasminogen KO mice over the two weeks with a significant reduction in lesion size between days 1 and 14 (*, *p*<0.05 by Kruskal-Wallis test with Dunn's post-hoc tests). **D.** H&E-stained vertical sections at the center of the liver lesion in WT (upper panels) and plasminogen-KO mice (lower panels). Arrowheads indicate granulation tissue, which was not observed in the KO mouse liver. Scale bars: 1 mm.

For Figs. 1, 2, 3, Two-way analysis of variance (ANOVA) with Bonferroni post-hoc tests and Kruskal-Wallis test with Dunn's post-hoc tests for multiple comparisons were performed by using Prism software (GraphPad Software Inc.). For Figs. S2 and S3, paired *t*-tests were performed. *P*<0.05 was considered statistically significant.

## Results

### Time-dependent changes in MCAO-induced cerebral infarction as revealed by micro-CECT

We first performed CECT using the MCAO mice, which are widely used as a model of cerebral infarction (n = 3). Using the optimized parameters (see Methods), we could non-invasively and sequentially examine cerebral infarction of the same MCAO mice from one day through two weeks after arterial occlusion (Fig. 1A). To assess infarction qualitatively and quantitatively, we measured the volume of the damaged area by setting a threshold of the CT value as described in Methods (Fig. 1B). On the first day after surgical occlusion (day 1), the infarct area was readily observed as LAA, indicating edema or a lack of penetration of the contrast agent due to a lack of blood flow in the affected area. In contrast, between days 3 and 7, HAA/(LAA+HAA) (%HAA) was increased, suggesting enhanced vascular permeability in the infarct area in the brain. To quantify these processes, we measured infarction volume in the areas of low and high attenuation and compared the %HAA values. As shown in Fig. 1B, lesion volume (LAA+HAA) decreased gradually over time until day 14. The LAA-indicating infarct area was predominant on day 1 (%HAA was 0 for all animals examined), but from day 7 through day 14 LAA was not detected and HAA was observed exclusively in the lesion (Fig. 1C, %HAA was 1 for all animals). To confirm the location and size of the infarct area, separate mice were sacrificed immediately following CECT image acquisition on selected days after the surgery, and histological analyses were performed (Fig. 1D, E). Figs. 1D and E are representative brain slices from MCAO mice at day 7, stained by TTC or treated by Evans blue, respectively. Consistent with the CECT images (Fig. 1A, F), infarction was observed in the right cerebral cortex (Fig. 1D). Leakage of Evans blue observed in the infarct area indicated increased vascular permeability in the region (Fig. 1E, S1), which is consistent with the increase of the HAA detected by CECT (Fig. 1A–C).

### The PIT-BD brain demonstrated similar changes in the damaged area comparable to the MCAO model

The PIT-BD mouse is another model for ischemic brain injury in which a certain area of the cerebral cortex is photochemically damaged to cause ischemia [3]. To test whether this model demonstrates a pattern comparable to that of the MCAO mice, we examined PIT-BD mice by CECT as described in Methods (n = 3, Fig. 2). Similar to the CECT images observed in the MCAO mice, the lesion area of the PIT-BD mice showed low attenuation on day 1 (Fig. 2A, B). On days 3 and 5, the %HAA gradually increased, and only HAA was observed on day 7 and 14. The volume of the lesion area (HAA+LAA) decreased over time in a manner similar to that in the MCAO mice (compare Fig. 1B and 2B). Constant elevation of the %HAA seen in MCAO (Fig. 1C) was also observed in the damaged area in the PIT-BD mice (Fig. 2C). Histological studies of the PIT-BD brains (Fig. 2D, E) demonstrated that, although the average volume of the infarction in the PIT-BD mice was significantly smaller than that of MCAO (compare Figs. 1B and 2B, also 1F and 2F), points of similarity in measure, such as increased permeability in the lesion, were observed between the MCAO (Fig. 1B) and PIT-BD models (Fig. 2B). The enhanced Evans blue leakage in the PIT-BD model (Fig. 2E, S1) was also consistent with the increase in the %HAA observed by CECT (Fig. 2B, C)

### Assessment of vascular permeability by Evans blue leakage

As we had observed elevated %HAA in the lesions of the MCAO and PIT-BD brains, we then measured the amount of Evans blue extravasated from the brain lesions, which is considered to reflect vascular (Blood-brain barrier) permeability (see Materials and Methods). After intravenous injection of the dye, we measured the amounts of the dye leaked into the ipsilateral hemisphere (including the lesion) as compared with those in the contralateral hemisphere (control) of the same animals (n = 5 for each animal model). As shown in Fig. S1, the average amount of dye extracted from the ipsilateral specimen (ipsi.) was significantly higher than that from the other half of the cerebrum (contra.) in both of the two animal models, indicating elevated vascular permeability in the lesion area of the brain.

### Differences in recovery from liver damage between wild-type and plasminogen-knockout mice

We performed CECT imaging studies of mice with ischemic liver injury, PIT-LD, and compared the effects of plasminogen (*Plg*) gene deficiency on liver injury and subsequent recovery (Fig. 3, S2; n = 4). The volume of the damaged area in the PIT-LD mice of the wild-type background (*Plg* +/+, WT) decreased over four days (Fig. 3A, B, S2A, C, E), and the lesion became undetectable by two weeks after the photoactivation (Fig. 3B). In contrast, in the plasminogen-knockout mice (*Plg* −/−, KO), the decrease of the lesion volume was slower than that of the WT controls (Fig. 3A, C, S2B, D, F). Two-way ANOVA with post-hoc tests for multiple comparisons revealed that there are significant differences in the lesion volumes between KO and WT mice on days 4, 7, and 14 (Fig. 3B; †, *P*<0.001, ‡, *P*<0.0001, §, *P*<0.0001). Of note, HAA was observed over the entire period of the study in mice of both genotypes, primarily in the marginal areas of the legion (Fig. 3A–C). Evans blue treatment demonstrated that the HAA at the border of the liver lesion overlapped with the region where intense Evans blue stain was observed (Figs. S2C and D, arrows). The difference in the lesion areas between WT and KO mouse liver was confirmed by histological analysis (Fig. 3D). Whereas the infarct area in the liver in WT mice decreased gradually, no significant change in the liver lesion area was observed in KO mice. High cellular layers (Fig. 3D, arrowheads), where the accumulation of macrophages, neutrophils and active hepatic stellate cells, collagen fiber deposition, and angiogenesis were previously reported [6], were observed in the WT, but not in the KO, mouse livers. Interestingly, despite this histological difference between the WT and KO mice, the HAA at lesion margins was detected by CECT in mice of both genotypes (Fig. 3A).

### Comparative volumetric analyses of the brain/liver lesions using CT and MRI

To examine whether the total lesion volumes of the MCAO, PIT-BD, and PIT-LD mice that we measured by CECT were accurate, we next performed comparative volumetric analyses of the same animals using micro-CT and micro-MRI (Fig. S3). The MCAO and PIT-BD mice (n = 4 each) on day 7 and the PIT-LD mice (n = 4) on day 4 were sequentially scanned by CT and MRI, and acquired images as shown in Fig. S3A were used for volumetric analyses as performed for the experiments shown in Figs. 1–3. Fig. S3B shows the lesion volumes of the three different model mice measured from CT and MR images. There was no significant difference in the lesion volumes calculated by the two different image acquisition techniques.

### Comparison of CECT and micro-MRI for the detection of stroke areas in the SHRSP brain

**Figure 4:**
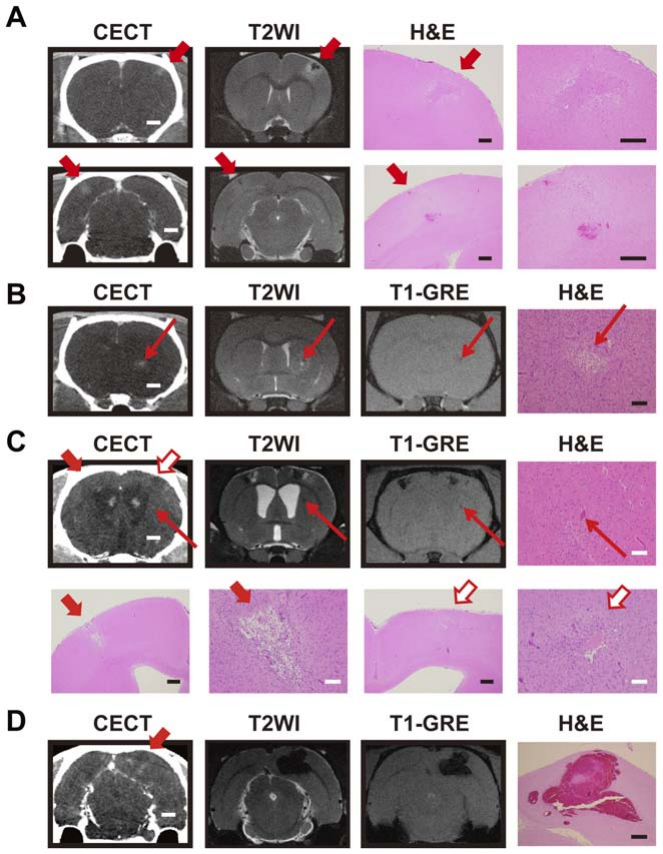
Comparisons of the CT and MR images of stroke lesions in the SHRSP rats. **A.** Images acquired with CECT (left), T2-weighted MR (T2WI, middle), and H&E staining (right) of the same stroke areas in an SHRSP rat brain two weeks after the onset. The areas indicated by arrows are suspected loci of hemorrhagic infarction. Infarct lesions with gliosis and inflammatory cell infiltration were observed in the upper and lower H&E images (left and right panels are the same lesion at low and high magnifications, respectively). Hemorrhage (or congestion) is also seen in the lower panels. Scale bars: 2 mm (CECT); 400 µm (H&E). **B.** Images acquired by CECT, T2WI, and T1-weighted gradient echo (GRE), along with an H&E image of identical stroke areas in an SHRSP rat brain after 3 weeks of stroke onset. Arrows indicate suspected hemorrhagic infarction of about 0.5 mm in diameter. Gliosis and small-sized cystic cavity formation are observed in the infarct area (H&E image). Scale bars: 2 mm (CECT), 100 µm (H&E). **C.** Images obtained with CECT, T2WI, T1-weighed GRE, and an H&E stain of identical stroke areas in an SHRSP rat brain 4 weeks after onset. Bold arrows (red and white) in upper panels indicate suspected hemorrhagic infarction. A suspected hemorrhage of about 0.1 mm in diameter was also detected (thin arrows). The corresponding areas were analyzed by H&E staining (upper right and lower panels). The lesion areas indicated by both red and white bold arrows in the H&E images are wedge-shaped infarcts with inflammatory cell infiltration, gliosis, and cystic cavities (lower panels at low and high magnifications). Scale bars: 2 mm (CECT), 500 µm (H&E, white bars) and 100 µm (H&E, black bars). **D.** Images of the posterior brain of the same SHRSP rat shown in C. The bold arrow indicates a suspected hemorrhagic infarction. Scale bars indicate 2 mm (CECT) and 500 µm (H&E, lower panel).

We next studied the SHRSP rats with CECT as a representative model of spontaneous brain disease. These rats exhibit individual differences in their symptoms, e.g., onset, severity, lesion size and localization. To determine whether images acquired by CECT have sufficiently high resolution and whether small infarction/hemorrhage in the brain can be detected as with micro-MRI, we compared the CT images of the brain from the same diseased SHRSP animals with the MR images (n = 5). As shown in Fig. 4, the spatial and contrast resolution of the ischemic lesion on micro-CT images was comparable to that of the micro-MR images (Fig. 4A–C). Fig. 4A shows images acquired from CECT, T2-weighted MR (T2WI), and H&E-stained section of the same stroke areas in an SHRSP rat brain at 2 weeks from the onset. Wedge-shaped HAAs were observed by CECT, suggesting increased permeability. The T2WI images of the corresponding lesion showed a low signal in the central region surrounded by a high-signal area, suggesting suspected hemorrhagic infarction. The H&E stained sections of infarct areas demonstrated gliosis and inflammatory cell infiltration either surrounded by a necrotic area (upper) or with partial hemorrhagic transformation (lower). Fig. 4B shows images acquired from CECT, T2WI, and T1-weighted gradient echo (GRE) along with an H&E stained section of the same stroke area in a different SHRSP rat brain after 3 weeks from the onset. HAA was detected by CECT, the co-existence of high and low signals was observed in the T2WI image, and low signal was observed in the GRE image, suggesting a suspected hemorrhagic infarction of about 0.5 mm in diameter. Histological analysis indicated cystic cavity formation with gliosis. Fig. 4C shows images from the same stroke areas in an SHRSP rat brain after 4 weeks from the onset. Wedge-shaped LAAs (Fig. 4C, red and white bold arrows) were observed by CECT, low and high signals were detected in T2WI, and low signal was observed in GRE, suggesting suspected hemorrhagic infarction. H&E images showed cystic cavity formation with gliosis and inflammatory cell infiltration (Fig. 4C, lower panels). We also detected a smaller lesion in the same rat brain (Fig. 4C, thin arrows) for which HAA in CECT and low signals in T2WI and T1-GRE were observed, suggesting hemorrhage. Histological analysis of the lesion confirmed hemorrhage of about 0.1 mm in diameter (H&E, upper panel). Fig. 4D demonstrates a different brain area of the same SHRSP rat as shown in Fig. 4C. LAA surrounded by HAA in CECT and low signals in T2WI and T1-GRE suggest hemorrhagic infarction, which was confirmed by H&E staining (Fig. 4D). In summary, we could detect infarction and/or hemorrhage in all rats we examined by either imaging method.

## Discussion

In this study, contrast-enhanced micro-CT was performed on different rodent models of ischemic/hypertensive diseases. We successfully detected brain injury or stroke in the mouse/rat brain with high spatial and contrast resolutions by continuous administration of a clinically-used iodinated contrast agent, Iohexol. Simple CT imaging and/or use of a bolus dose injection of contrast agent are not suitable for brain and liver imaging in rodents due to the low contrast resolution of micro-CT scans and rapid excretion of the contrast agent in these species (Fig. S4). It should be noted that the high infusion rates of the contrast agent did not cause lethality or severe damage to the animals used in this study. Thus, the CECT methods we describe here are practical for soft tissue imaging, including respiratory-gated liver image acquisition and brain imaging. The low attenuation areas (LAA) found in the ischemic brain on day 1 by CECT were consistent with the infarct area detected by histology. In addition, our CECT study using two independent ischemic mouse models also demonstrated that the LAA decreased from day 3, becoming undetectable by day 7, and were gradually replaced by HAA. The volumetric measurements by CECT were comparable with those by MRI, suggesting that the quantitative analyses based on the images acquired from CECT are accurate and reliable (Fig. S3). Furthermore, the changes in vascular permeability were confirmed by the measurement of Evans blue dye leakage. These data suggest that lesion volume and the degree of vascular permeability of the infarct area can be assessed by measuring lesion volume or %HAA using our CECT methods. We also demonstrate by comparison of the images acquired from micro-CT and micro-MRI (11.7 T) from the rat stroke model that CECT can detect infarct or hemorrhagic areas at a resolution of less than 0.5 mm in diameter, which is comparable to the detection limits of the high-end micro-MRI. We further performed comparative volumetric analyses using CECT and MRI, demonstrating that average lesion volumes measured by the two different image acquisition techniques are comparable with each other. The data suggest that volumetric measurement using our CECT methods is quite reliable.

Our study also demonstrates that sequential monitoring of the same animal over time with all these animal models can reveal quantitative and qualitative alterations of lesions, suggesting that this non-invasive, serial approach is beneficial and efficient for studying pathological changes *in vivo*. For instance, the volumes of the lesion areas (LAA+HAA) in the MCAO and PIT-BD mice gradually decreased, and %HAA was elevated until LAA became undetectable after one week (Figs. 1, 2). The data also suggest that vascular permeability is increased in the infarct area for the first week, followed by a decrease in the volume of damaged area over time. Consistent with the previous studies [5], we have also demonstrated that, while the lesion volume of the PID-LD liver in WT mice decreased over time, the volume of the liver lesion in the plasminogen KO mice was not significantly reduced (Fig. 4). The ischemic liver lesion was surrounded by HAA, which was manifest in the majority of the lesion areas from day 1 through day 14 in both the plasminogen KO and WT animals. It has previously been reported that, in the marginal region of the liver lesions in WT animals, inflammatory cell and fibroblastic cell infiltration, as well as neovascularization, is observed [5], [6]. Although these normal biological responses are not observed in the plasminogen KO mice, our CECT data demonstrating HAA in the KO mice liver lesions imply qualitative changes in the damaged area, such as increased vascular permeability or congestion.

The MCAO and PIT-BD mouse brain images acquired from CECT demonstrated similar patterns in temporal changes in the ischemic areas, where average lesion volume (LAA+HAA) declined and %HAA increased, indicating that vascular permeability progressively increased during the first week of observation (Figs. 1, 2). The data presented here have demonstrated not only that these quantitative and qualitative evaluations are consistent with those previously reported, but also that we could concurrently compare the two independent brain ischemia models by imaging several animals in a few hours. In addition, such experiments can also be applied to monitoring groups of inbred animals that show, for instance, individual variation in phenotypes or varying responses to drug candidates. Given that analogous pathological trends were observed in the two ischemic models as well as in humans, our data support the view that these mice are good models for human ischemia.

Our comparative study of micro-CT and micro-MRI using the SHRSP stroke model has demonstrated that small hemorrhage or infarction in the brain (0.5 mm or less in diameter) can be detected by the CECT (Fig. 4B, C). The data suggest that the spatial and contrast resolutions of the micro-CT images of the brain are sufficient for analyzing strokes in this small rodent model. By contrast, micro-MRI is more informative for diagnosis of stroke, as different clinical phases (super acute, acute, subacute, or chronic) can be precisely determined. However, issues with current micro-MRI are: i) it is relatively costly, ii) it requires special skill for parameter settings, and iii) it requires a facility for a high magnetic field. In contrast, the micro-CT does not have any restrictions for installation or requirements for a radiation shield. In addition to its cost-effectiveness and easy-to-use features, micro-CT also provides high-resolution images and rapid data acquisition capabilities [1], which should encourage the use of micro-CT in future studies.

In summary, our present study has demonstrated the utility and the advantages of micro-CT in studying pathological animal models. First, this non-invasive *in vivo* imaging in small animals facilitates longitudinal monitoring of individual animals in experiments. Therefore, this method can reduce number of animals need for such studies and accelerate the pathophysiological understanding of disease processes and screening of therapeutic compounds. Second, micro-CT imaging provides precise, reliable, and detailed information on pathological changes in soft tissues. As shown in this study, the results obtained by imaging were consistent with those from conventional histological studies. In addition, repeated monitoring over time of the same animals would enable accurate quantitative and qualitative studies of particular lesions. Third, this non-invasive approach would be valuable for preclinical research. For instance, longitudinal micro-CT monitoring is advantageous for preclinical research, as this approach cannot be applied in human clinical studies due to high radiation doses or other ethical issues. Thus, further translational studies on pathological models using the micro-CT technique would also provide knowledge for preemptive medicine.

## Supporting Information

Figure S1 **Increased vascular permeability in the MCAO and PIT-BD mouse lesions.** Evans blue dye was injected intravenously and average amounts of extravasated dye were calculated for brain slices from injured (ipsilateral) and control (contralateral) hemispheres of the MCAO and PIT-BD cerebra. The difference between the ipsilateral (ipsi.) and contralateral (contra.) hemispheres was considered to represent the amount of extravasated Evans blue, reflecting increased vascular permeability in the infarct area. *** *P*<0.001; **P<0.01 (paired-*t* test).
(TIF)

Figure S2 **Histological analysis of the PIT-LD mouse liver.** **A, B.** Liver surfaces of the photochemically-induced liver damage in the WT (A) and the plasminogen KO mice (B) on days 1 and 7. Whereas the lesion volume was dramatically diminished within one week in the WT liver, no significant change was observed in the KO liver, as indicated in Fig. 3C, D. Scale bars: 1 mm. **C, D.** For evaluation of vascular permeability, Evans blue administration was performed on the PIT-BD livers from WT and KO mice. Two weeks following the surgical procedure, the recovery process was observed in the WT liver, but not in the KO liver. Note that the marginal area of the lesions (arrows) was densely stained regardless of the genotype, and, consistent with the HAA surrounding liver damage observed by CECT, dark staining was continuously observed in the lesion area of the KO liver after 2 weeks (See Figure 3A). Scale bars: 1 mm. **E, F.** H&E-stained images of the liver injury on days 7 and 14. Scale bars: 1 mm.
(TIF)

Figure S3 **Comparative volumetric analyses of the brain/liver lesions using CT and MRI.** **A.** Representative images of the brain or liver lesions of the same animals sequentially acquired by CECT and MRI. **B.** Comparison of lesion volumes of the same animals analyzed by two different image acquisition techniques. Lesion volumes of the three different animal models calculated from CECT (left, CT) and MR (right, MRI) images are shown (n = 4 each). There was no significant difference in the lesion volumes between CECT and MR images in either model (paired-*t* test, *P*>0.05).
(TIF)

Figure S4 **Comparison of brain CT images of the same animals acquired with or without contrast agents.** CT images of the same MCAO (upper) and PIT-BD (lower) mouse brains acquired by simple CT (CT, left) or contrast-enhanced CT (CECT, right). It is of note that although faint signals in the ischemic areas of the brain are observed in the CT images, the areas are significantly smaller than those detected by CECT.
(TIF)
