# Supplementary figures and images for: In Vivo Diagnostic Imaging Using Micro-CT: Sequential and Comparative Evaluation of Rodent Models for Hepatic/Brain Ischemia and Stroke
Source: PLoS One. 2012 Feb 23;7(2):e32342. doi: 10.1371/journal.pone.0032342 (PMC3285673; doi:10.1371/journal.pone.0032342)

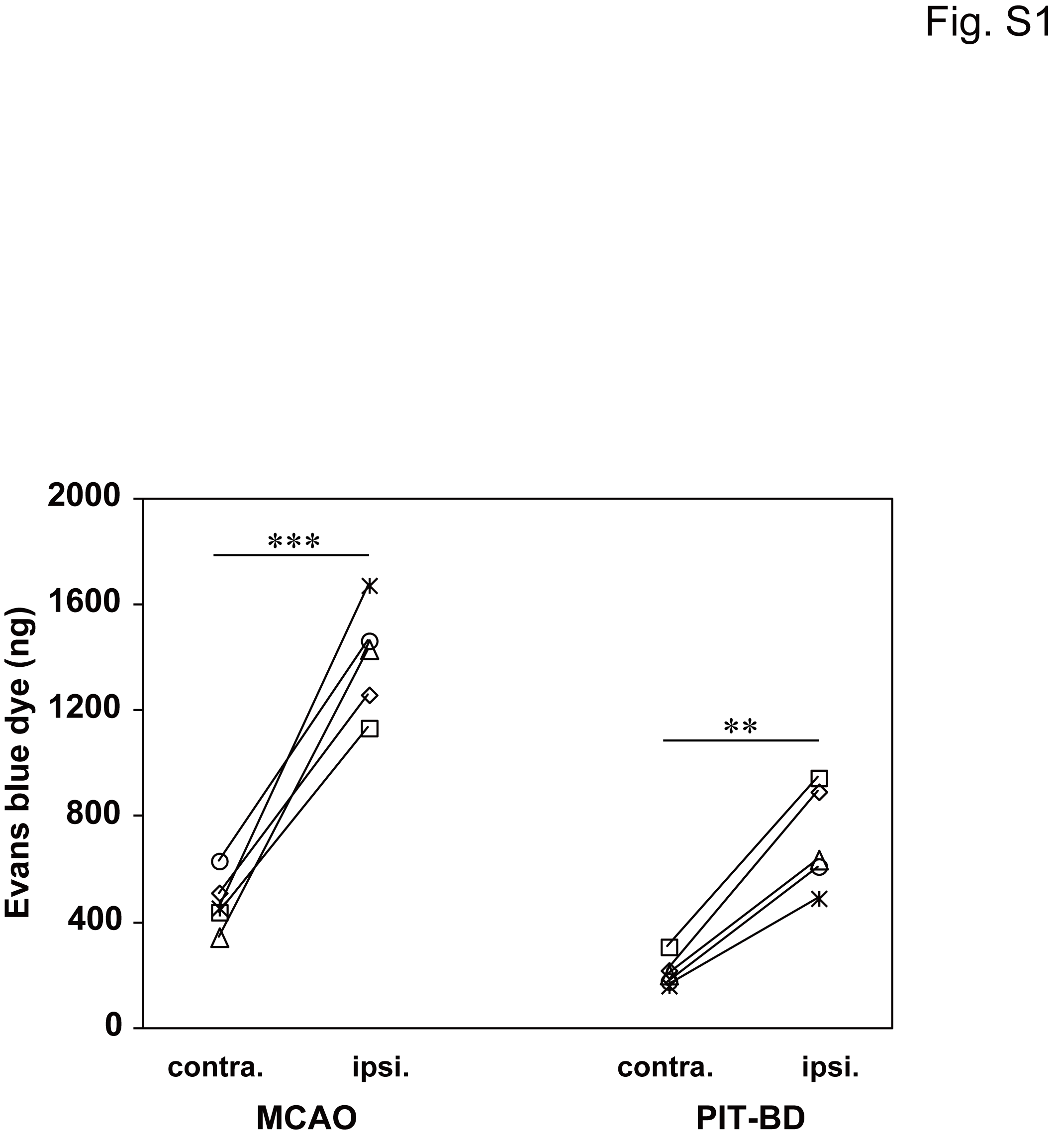

Supplement: Figure S1 — Increased vascular permeability in the MCAO and PIT-BD mouse lesions. Evans blue dye was injected intravenously and average amounts of extravasated dye were calculated for brain slices from injured (ipsilateral) and control (contralateral) hemispheres of the MCAO and PIT-BD cerebra. The difference between the ipsilateral (ipsi.) and contralateral (contra.) hemispheres was considered to represent the amount of extravasated Evans blue, reflecting increased vascular permeability in the infarct area. *** P<0.001; **P<0.01 (paired-t test). (TIF) [file pone.0032342.s001.tif]

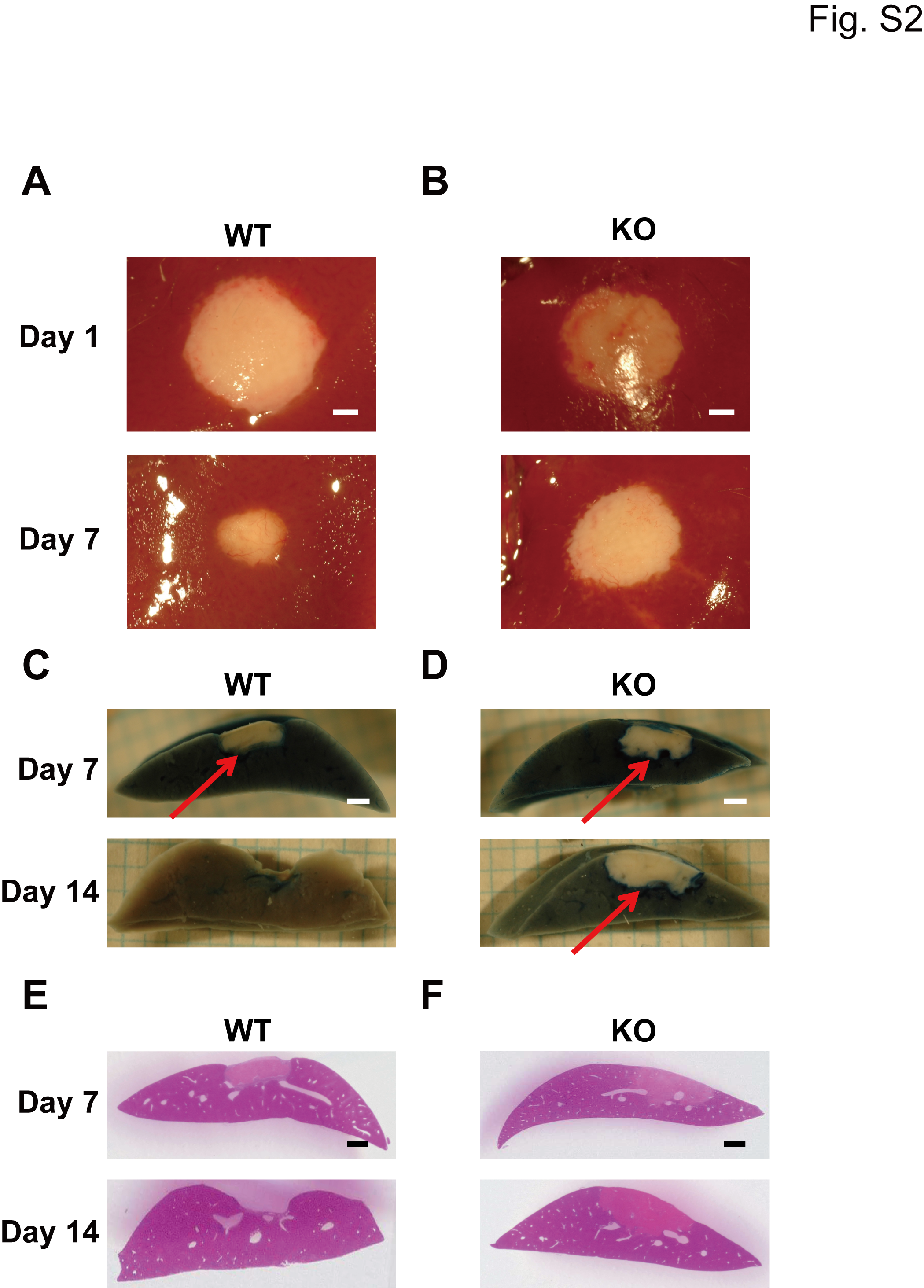

Supplement: Figure S2 — Histological analysis of the PIT-LD mouse liver. A, B. Liver surfaces of the photochemically-induced liver damage in the WT (A) and the plasminogen KO mice (B) on days 1 and 7. Whereas the lesion volume was dramatically diminished within one week in the WT liver, no significant change was observed in the KO liver, as indicated in Fig. 3C, D. Scale bars: 1 mm. C, D. For evaluation of vascular permeability, Evans blue administration was performed on the PIT-BD livers from WT and KO mice. Two weeks following the surgical procedure, the recovery process was observed in the WT liver, but not in the KO liver. Note that the marginal area of the lesions (arrows) was densely stained regardless of the genotype, and, consistent with the HAA surrounding liver damage observed by CECT, dark staining was continuously observed in the lesion area of the KO liver after 2 weeks (See Figure 3A). Scale bars: 1 mm. E, F. H&E-stained images of the liver injury on days 7 and 14. Scale bars: 1 mm. (TIF) [file pone.0032342.s002.tif]

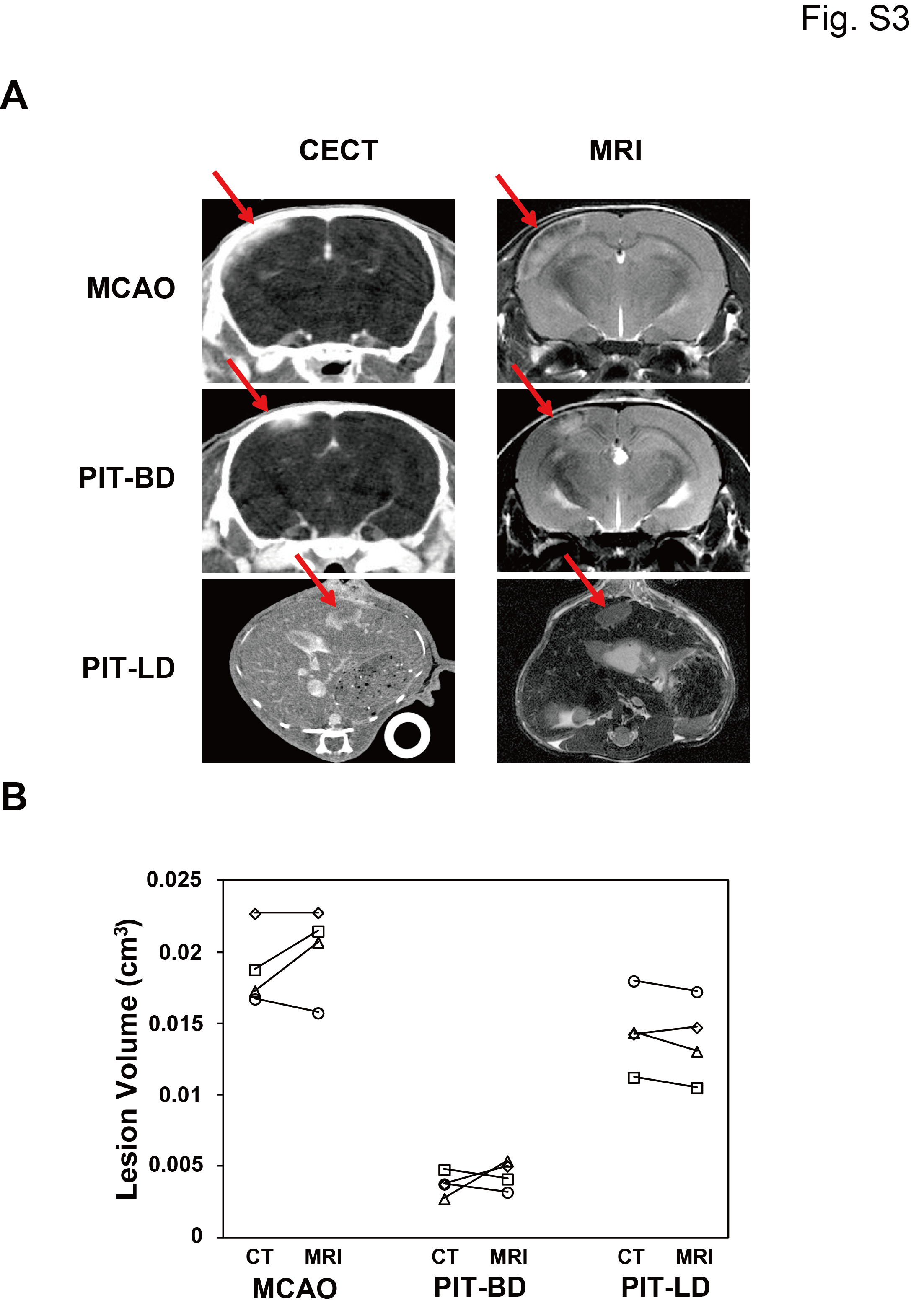

Supplement: Figure S3 — Comparative volumetric analyses of the brain/liver lesions using CT and MRI. A. Representative images of the brain or liver lesions of the same animals sequentially acquired by CECT and MRI. B. Comparison of lesion volumes of the same animals analyzed by two different image acquisition techniques. Lesion volumes of the three different animal models calculated from CECT (left, CT) and MR (right, MRI) images are shown (n = 4 each). There was no significant difference in the lesion volumes between CECT and MR images in either model (paired-t test, P>0.05). (TIF) [file pone.0032342.s003.tif]

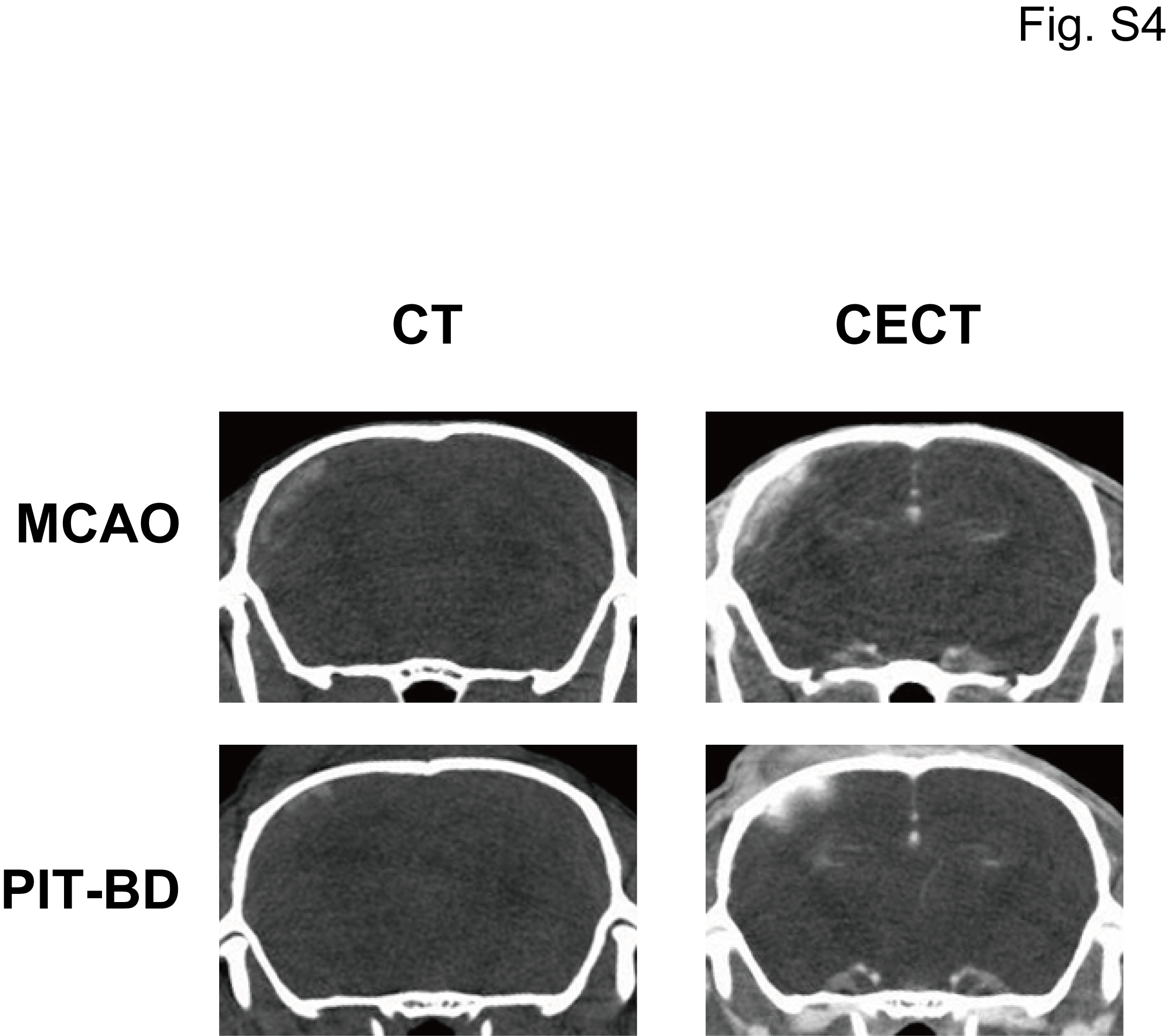

Supplement: Figure S4 — Comparison of brain CT images of the same animals acquired with or without contrast agents. CT images of the same MCAO (upper) and PIT-BD (lower) mouse brains acquired by simple CT (CT, left) or contrast-enhanced CT (CECT, right). It is of note that although faint signals in the ischemic areas of the brain are observed in the CT images, the areas are significantly smaller than those detected by CECT. (TIF) [file pone.0032342.s004.tif]
